# Bilateral corpus callosum and corona radiata infarction due to cerebral venous sinus thrombosis presenting as headache and acute reversible aphasia: a rare case report

**DOI:** 10.1186/s12883-020-01829-7

**Published:** 2020-06-19

**Authors:** Rui Lan, Yun-zhi Ma, Xiao-ming Shen, Ji-tao Wu, Chun-qing Gu, Yong Zhang

**Affiliations:** 1 Encephalopathy Hospital, The First Affiliated Hospital of Henan University of Chinese Medicine, Zhengzhou, 450000 Henan China; 2 Cerebral Palsy Rehabilitation Department, The Third Affiliated Hospital of Zhengzhou University, Zhengzhou, 450000 Henan China

**Keywords:** Cerebral venous sinus thrombosis, Corpus callosum and radiata infarction, Headache, Acute reversible aphasia, Case report

## Abstract

**Background:**

Cerebral venous sinus thrombosis (CVST), a rare cause of cerebral infarction, is often unrecognized at initial presentation. We report the case of a patient with bilateral corpus callosum and corona radiata infarction due to cerebral venous sinus thrombosis presenting as headache and acute reversible aphasia.

**Case presentation:**

A 30-year-old female patient presented with headache, vomiting, and motor aphasia. She was 20 days post-partum and had a lower than normal food intake following a normal vaginal delivery. Brain magnetic resonance images revealed a bilateral corpus callosum and corona radiata infarction. MR venography (MRV) and digital subtraction angiography (DSA) images showed a signal void in the anterior aspect of the superior sagittal sinus and inferior sagittal sinus, ophthalmic vein expansion, and the reversed direction of venous flow. In addition, images showed non-visualization of the left transverse sinus. The left slender sigmoid sinus and small internal jugular vein were also noted. The diagnosis of cerebral venous thrombosis was considered based on the above findings. The patient was managed with anticoagulation therapy, and recovered substantially after treatment.

**Conclusions:**

Bilateral corpus callosum and corona radiata infarction is very rare. However, for patients who clinically show cranial hypertension and neurological deficits during the puerperium period, the possibility of CVST should be considered. Furthermore, DSA plays an important role in the diagnosis of CVST, and should be routinely checked. Early diagnosis is crucial for the patient suffering from CVST.

## Background

Cerebral venous sinus thrombosis (CVST) is a serious disorder with an annual incidence estimated to be 3–4 cases per million [1]. One study found that causes of CVST include congenital or acquired diseases, such as hematological disorder infection or dehydration [2]. CVST patients commonly present with a variety of clinic presentations such as headache, vomiting, neurological deficits, encephalopathy, and seizures [3] .

To our knowledge, hypercoagulable states such as pregnancy, puerperium, some malignancies, and the use of oral contraceptives may significantly increase the risk of thrombosis. Anticoagulation is the primary treatment for patients with CVST. Additionally, local infusion of recombinant tissue plasminogen activator and mechanical thrombectomy are used in the treatment of CVST [4]. Early diagnosis and prompt anticoagulation therapy is key in successful management.

Herein, we describe a rare case of a young mother in the postpartum period. She showed cranial hypertension symptoms and reversible aphasia due to bilateral corpus callosum and corona radiata infarction resulting from CVST. After anticoagulation treatment, abnormal findings on MR images were essentially reversed.

## Case presentation

A 30-year-old postpartum Han-Chinese woman presented to the department with gradually worsening headache and vomiting for 10 days followed by acute motor aphasia. She was 20 days post-partum and had a decreased appetite following a normal vaginal delivery. The pregnancy was healthy with no complications. The patient had no medical history of hypertension, diabetes mellitus, coronary heart disease, or pulmonary tuberculosis. There was no family history.

On presentation, the patient (height 165 cm, weight 60 kg, body mass index 22 kg/m^2^) was drowsy, and unable to cooperate perfectly. Neurological examinations showed motor aphasia. Basic observations revealed: heart rate of 92 beats/min, blood pressure of 96/50 mmHg, temperature of 36.6 °C and blood glucose of 5.1 mmol/L. Laboratory findings including routine blood and urine tests, blood chemistry analysis, autoimmune markers, and homocysteine level were normal; prothrombin time of 14.1 S, international normalized ratio (INR) of 1.17, activated partial thromboplastin time of 48.3 S, fibrinogen of 4.52 g/L, thrombin time of 17.6 S, and D-dimer of 1.03 mg/L. Additional extensive research was further performed: erythrocyte sedimentation rate of 15 mm/h, C-reactive protein of 13.6 mg/L, protein C of 71%, protein S of 76%, and lupus anti-coagulant screening of 1.12%. Screening for anti-cardiolipid, anti-nuclear, anti-keratin, anti-SSA/Ro, anti-SSB/La, perinuclear anti-neutrophil cytoplasmic, and cytoplasmic anti-neutrophil cytoplasmic antibodies were negative. Anti-double-stranded-DNA antibody was 55.22 IU/mL, anti-SM-D1 antibody was 1.97 RU/mL, anti-cyclic citrullinated peptide antibody was 3.89 RU/mL, anti-RA33 antibody was 5.54 U/mL, rheumatoid factor IgG was 10.12 RU/mL, rheumatoid factor IgA was 2.12 RU/mL, and rheumatoid factor IgM was 14.63 RU/mL.

**Fig. 1:**
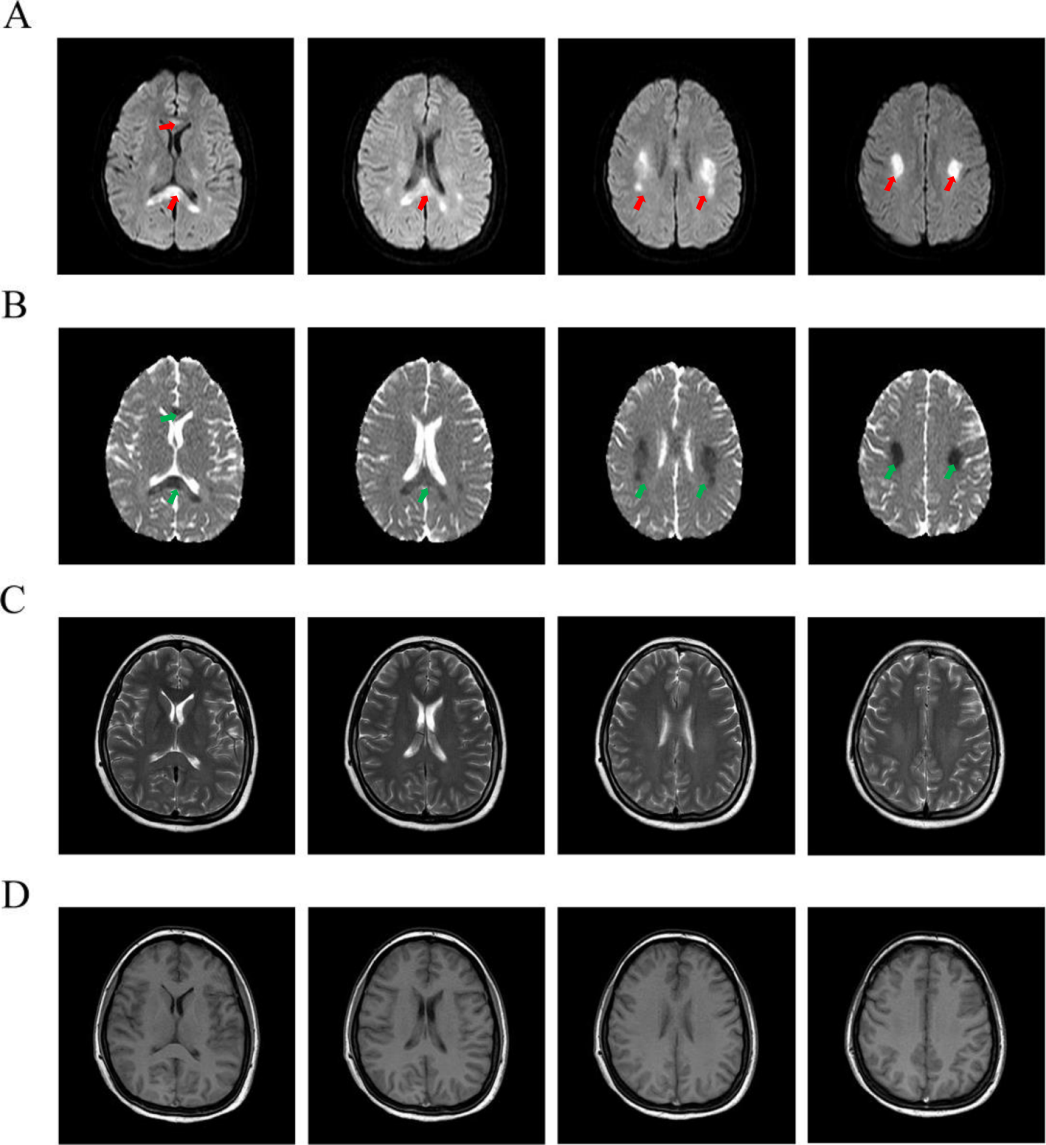
Axial MRI images of brain. (**a**) DWI images showed high signal intensity involving corpus callosum and radiata (red arrows). (**b**) ADC images indicated that the signal intensity was opposite to the DWI images in the same lesions (green arrows). T2-weighted axial images (**c**) and T1-weighted images (**d**) were normal

Lumbar puncture was performed immediately after admission and the opening pressure of cerebrospinal fluid (CSF) was 45 cm H_2_O. CSF analysis showed 2 lymphocytes per mm^3^, glucose was 80 mg/dL, total protein was 125 mg/dL, and Cl^−^ was 436.035 mg/dL. Further cerebrospinal fluid culture and virus tests were negative. An urgent brain magnetic resonance imaging (MRI) revealed bilateral corpus callosum and radiata infarction (Fig. 1). Diffusion-weighted imaging (DWI) showed sharply delineated areas of diffusion restriction involving corpus callosum and corona radiate, which was consistent with ADC images. Interestingly, the infarction areas were bilateral.

**Fig. 2:**
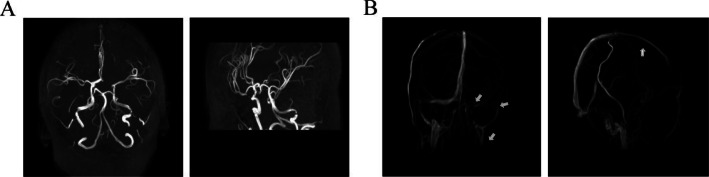
Brain MRA and MAV images. (**a**) The MRA images were normal. (**b**) The MRV images indicated a signal void in the transverse aspect of the superior sagittal sinus, non-visualization of the left transverse sinus, and small sigmoid sinuses and jugular vein (white arrows)

MR angiography (MRA) showed cerebral arteries were normal, but MR venography (MRV) revealed a signal void in the transverse aspect of the superior sagittal sinus and non-visualization of the left transverse sinus. Small sigmoid sinuses and jugular vein were also noted (Fig. 2). When the clinical profile, the results of the above mentioned laboratory tests, plus CSF and MRI images were combined, we suspected a diagnosis of CVST.

**Fig. 3:**
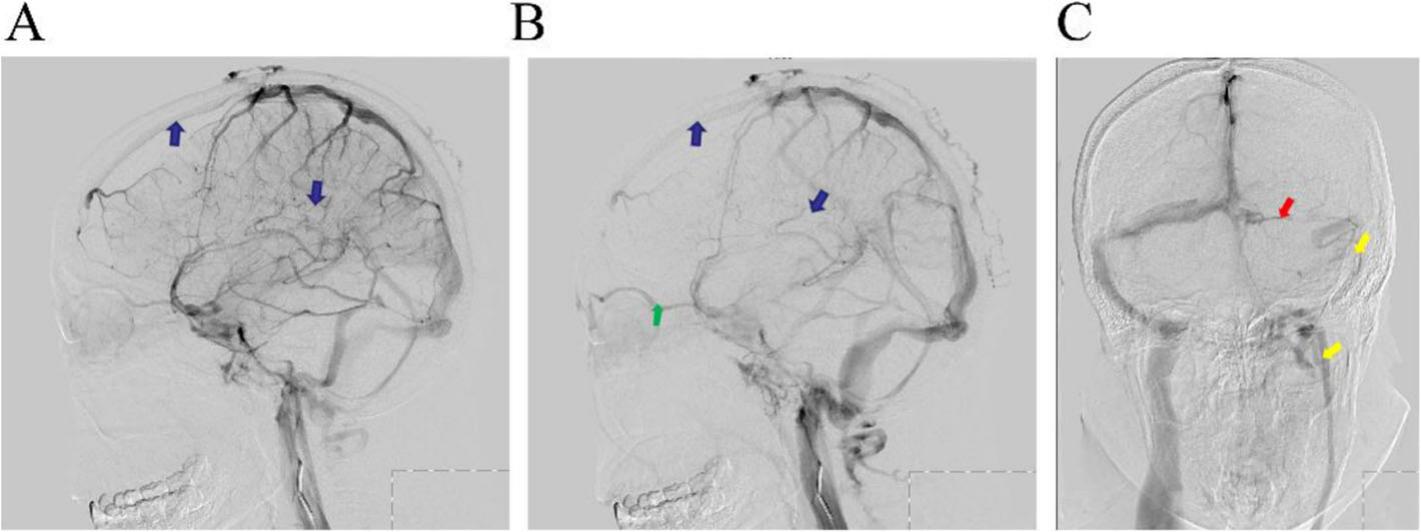
The DSA images indicated a signal void in the anterior aspect of the superior sagittal sinus, lack of flow within the inferior sagittal sinus (**a, b**, blue arrows), ophthalmic vein expansion, and the reverse direction of the venous flow (**b**, green arrow). **c**: Non-visualization of the left transverse sinus (red arrow). Slender sigmoid sinus and small internal jugular vein are indicated by yellow arrows

Digital subtraction angiography (DSA) was performed to verify our diagnosis, and the images revealed poor filling in the superior and inferior sagittal sinus. In addition, we found ophthalmic vein expansion, reverse direction of the venous flow, and non-visualization of left transverse sinus. A slender sigmoid sinus as well as small internal jugular vein were noted (Fig. 3).

**Fig. 4:**
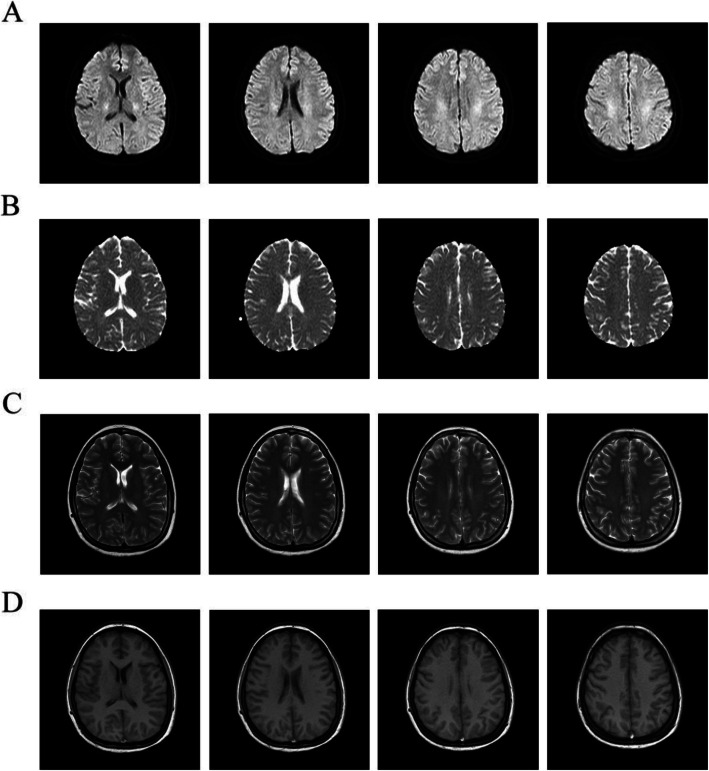
The post-intervention brain MRI. The DWI (**a**) and ADC (**b**) images showed minimal abnormal signal at the corpus callosum and radiate. No abnormality was found in T2-weighted axial images (**c**) and T1-weighted images (**d**)

Based on the above findings, the diagnosis of CVST with venous infarction was considered likely. The patient was managed with anticoagulation (oral warfarin and injection low molecular weight heparin) and anti-edema measures (glycerin fructose) immediately upon diagnosis confirmation. The treatment was effective and resulted in marked recovery from clinical symptoms within 1 week and consequently INR reached the target range of 2–3. After 1 week, the patient was symptom-free and follow-up MRI revealed reversible changes in DWI and ADC (Fig. 4). With anticoagulation therapy continuing, she was discharged with close follow-up. Two and a half years later, she was stable.

## Discussion and conclusions

CVST is difficult to diagnose due to its variable clinical signs and symptoms. The clinical features of CVST include headache, focal deficits, seizures, impairment of consciousness, in various combinations and degrees of severity [5]. One study found that the neurological symptoms and signs at admission were central motor or sensory deficits, aphasia and other neuro-deficits (40–60%), syndrome of isolated intracranial hypertension with headache, vomiting and blurred vision owing to papilledema (20–40%) and impaired consciousness (10–20%) [6].

Hereditary thrombophilia, pregnancy and puerperium, acquired hypercoagulable and hyperviscosity states, sinus trauma, regional infection, spinal anesthesia, medical/surgical conditions, malignancy, systemic illness, infection, coagulopathy, medication such as oral contraceptive drugs, and hormone replacement therapy are common risk factors of CVST [5, 7, 8]. The pathophysiology of pregnancy and the postpartum period is thought to be related to significant hemodynamic changes during pregnancy and after delivery [9]. One study indicated that an elevated risk of thrombosis persisted until at least 12 weeks after delivery, however, the absolute increase in risk beyond 6 weeks after delivery was low [10]. CVST is the most common cerebrovascular incident during the puerperium. Our patient presented with severe headache and vomiting as an initial presentation due to intracranial hypertension, and she quickly developed motor aphasia.

Cerebrovenous reflux disorders and CSF absorption disorders are the main pathological changes of CVST. When the sinuses involve many collateral veins, or the thrombus expands to the cerebral cortical veins, will result in increased intracranial pressure and cerebrovenous and CSF circulation disorders, and further lead to cerebral edema, cerebral hemorrhage, or necrosis. Severe intracranial hypertension and venous blood stasis, which could not be corrected in time, will further affect arterial blood flow and lead to cerebral ischemia, hypoxia, and even infarction. Radiological examination plays a crucial role in the diagnosis of CVST. Brain MRI and MRV, enable direct visualization of the thrombus in the affected cortical vein, and are used, when available, for the diagnosis and follow-up of CVST. Loss of signal-void appearance on an MRI is the main finding in cases with CVST. DSA still is the gold criteria for diagnosis of CVST. In our case, focal neurological deficits and intracranial hypertension were the first clues. The DWI images indicated that high signal intensities in the bilateral corpus callosum and radiata, were opposite from signals in ADC images. However, cerebral arteries were normal. The MRV and DSA images demonstrated a signal void in the anterior aspect of the superior sagittal sinus and lack of flow within the inferior sagittal sinus, which was the cause of bilateral cerebral infarction in the drainage areas of the involved cerebral venous sinus.

In the acute treatment of CVST, systemic anticoagulant therapy containing heparin and warfarin are recommended. When a patient presents with neurological deficit deterioration or coma, is nonresponsive to medical treatment, or there is evidence of a severe mass effect or intracranial hemorrhage, then decompressive hemicraniectomy or endovascular therapy including thrombolysis and mechanical thrombectomy should be performed [4]. In the present study, the patient was treated with anticoagulant therapy to stop the thrombotic process and fully recovered.

For women presenting with neurological deficits during pregnancy or puerperium, we should consider CVST and perform a series of screening examinations. MRV or DSA imaging should be carried out as soon as possible. Early diagnosis and restoration of blood flow is crucial for a good outcome.

## Data Availability

All data generated and analyzed in this study are included in this article.

## Abbreviations

CVST: Cerebral venous sinus thrombosis
MRV: Magnetic resonance venography
DWI: Diffusion-weighted imaging
DSA: Digital subtraction angiography
CSF: Cerebrospinal fluid
MRA: Magnetic resonance angiography
INR: International standardized ratio
